# Response to novelty induced by change in size and complexity of familiar objects in Lister-Hooded rats, a follow-up of 2019 study

**DOI:** 10.1038/s41598-021-89289-y

**Published:** 2021-05-13

**Authors:** Wojciech Pisula, Klaudia Modlinska, Anna Chrzanowska, Katarzyna Goncikowska

**Affiliations:** Institute of Psychology, Polish Academy of Sciences, Warsaw, Poland

**Keywords:** Behavioural ecology, Psychology, Reward, Animal behaviour

## Abstract

This study examines the relationship between the change in size and change in complexity of well-known/familiarized objects and exploratory activity regulation in rats. In our experiment, the rats were exposed to three types of environmental novelty in a well-familiarized chamber: (1) addition of new tunnels to the chamber, (2) increased size of a familiarized tunnel, and (3) increased complexity of the existing tunnels. The animals responded to the addition of new tunnels with a significant behavioural shift involving increased exploration of the newly installed tunnels. This effect was stable across all three test trials. The rats exposed to a change in size of the familiar object initially reacted with a behavioural shift towards the enlarged tunnel but then re-focused on the unchanged one. There was also a significant increase in the frequency of moving between the zones of the chamber. The experimental group exposed to an increased complexity of familiar objects responded with a pronounced behavioural shift towards the complex tunnel and then slightly intensified their exploration of the unchanged one. A decrease was also observed in the frequency of moving between the zones of the chamber in the first and second test trials. In the effect size analysis, no differences were found in any of the three groups, which suggests that all manipulations had similar impact. The data obtained in this study supports the view that in rats, curiosity is at least two-dimensional: activational and cognitive. The activational aspect of curiosity may be explained by novelty-related arousal processes, while the cognitive processes are activated at longer time intervals in response to more complex stimulation. The validation of this hypothesis requires further research involving manipulations with a recently standardized protocol for measuring free exploration.

## Introduction

This study is a direct follow-up of our previous paper^1^, in which we sought to analyse the relationship between the responses to novelty (defined as a change occurring in a well-familiarized environment) and different types of change. We used an experimental chamber to measure exploratory behaviour which we also described in detail in our paper^1^, and we followed a behavioural measurement protocol that we had recently developed^2^. The chamber was fitted with configurable objects, enabling diverse, yet strictly controlled, re-arrangements of the elements in the test environment. In our earlier study^1^, three types of environmental manipulation were applied: (1) adding new objects to the experimental chamber, (2) removing some of the objects; and (3) reducing the complexity of the objects present in the chamber. It was demonstrated that the rats responded to the introduction of new objects with a pronounced behavioural shift towards the newly installed objects. This effect was manifested by a significant increase in the time spent in the changed zone of the chamber, as well as the amount of time spent on contact with the modified objects in the changed chamber zone. Consequently, the rats spent less time in the unchanged zone, less time on contact with the objects in this zone, and they stayed longer in the starting box. There was also a decrease in the frequency of moving between the zones and the frequency of contact with the objects in the unchanged zone of the chamber. The rats from the group exposed to the removal of objects from the chamber barely responded to that experimental manipulation. The only response observed was their longer stay in the starting box. The rats exposed to the reduction in environmental complexity, responded to the experimental manipulation in a complex way. In the first test trial, there was an increase in the duration and frequency of contact with the objects in the changed zone. However, in the third test trial, the rats spent more time in the unchanged zone, as well as on contact with the objects in that zone.

The results of the 2019 study^1^ allowed us to differentiate between the “increase” vs “decrease” manipulation effects. It is clear that the rats responded to the addition of new objects in a more pronounced way than to the removal of the existing objects or reduction in their complexity. Those results may be illuminated from an ecological psychology standpoint. An addition of new objects creates a new environmental quality that may be described as ‘affordance-inviting’^3^. What was still unclear, however, was the effect of the addition of a new object itself. The manipulation involved both an increase in the complexity of the new environmental characteristics and an increase in the size of the objects. Therefore, on the basis of the previous study^1^, it was impossible to state which of the object properties changed: complexity or size. This issue, nonetheless, is of crucial importance from the point of view of the animal’s cognitive system.

In their review of neophobia tests, Greggor, Thornton and Clayton^4^ formulated several guidelines for studying animal behaviour. They claimed that novelty increases with stimulus complexity such as patterns, colours, textures. This statement is patently true and valid. However, it may lead to researchers erroneously ignoring other sources of novelty to which animal brains might be sensitive. One of the best and most widely accepted definitions of novelty is that put forward by Bevins^5^ [p 189]: novelty is ‘a change in stimulus conditions from previous experience’. This definition is an outcome of a long research tradition associated mainly with Daniel Berlyne^6,7^, in which novelty was regarded to be a result of interactions between an individual’s experience and actual input. Therefore, there are strong theoretical grounds for investigating responses to novelty in a wider ecologically valid context that involves any change of stimulus. Even though the role of environmental complexity in shaping rat behaviour and preference for complexity has been widely recognized^1,8,9^, the behavioural effects triggered by more fundamental environmental changes (e.g. change in the size of familiar objects) have not yet been explored in any depth. With this study, we sought to examine the relationship between the change in size and change in complexity of well-known/familiarized objects in the experimental chamber. To this end, we applied three manipulations of which the first, the addition of new tunnels, has already been described in detail in our previous study^1,2^. The animals exposed to that manipulation served as a reference group for the present study. The two manipulations applied in our experiment involved a change (increase) in object size in the first experimental group, and an increase in complexity in the second experimental group.

## Methods

### Animals

The experiment was conducted on the rat model^10^. The sample consisted of 40 experimentally naive male Lister Hooded rats. The animals were sourced from Charles River, Germany, via AnimaLab Sp. z o.o., Poland. The rats were housed in the vivarium of the Institute of Psychology, Polish Academy of Sciences, Warsaw, Poland. At the onset of the study, the rats were approx. 80 days old and weighed approx. 250 g.

The rats were housed in groups of 3–4 in Tecniplast© Eurostandard Type IV cages 610 mm × 435 mm × 215 mm with dust-free softwood granules Tierwohl Super© as bedding and with ad libitum access to water and standard laboratory fodder Labofeed H, WP Morawski, Kcynia, Poland. The day/night cycle was set at 12/12 h, with the lights-on at 8.00 a.m., the temperature was maintained at a constant 21–23 °C, and humidity at 45–60%. Prior to the experiment, the cages were cleaned once a week. However, in order to ensure that the experimental procedure was not disrupted, the cages in which the test animals were kept were cleaned just before the onset of the experiment and again after the end of the experiment.

All the rats were housed, bred and taken care of in accordance with the Regulation of the Polish Minister for Agriculture and Rural Development of 14 December 2016 on laboratory animal care. The experimental procedures had been approved by the First Local Committee for Ethics in Animal Experimentation in Warsaw, Poland, permit #756/2018.

The sample size was estimated using a commonly used formula for calculating sample size for repeated measures^11^:

$$ {\text{N }} = { 2 } + {\text{ Cs}}/{\text{d}}^{{2}} $$

where: s—standard deviation of population means, d—difference in means − the effect size, C—constant dependent on the value of α significance level and 1-ß power.

For the purpose of our study, we used the following parameters: α = 0.05; β = 0.20 C = 10.51. Group size calculations were based on our previous study^1^, in which the average time spent on exploring the changed objects was M = 125.3, with standard deviation s = 32, and on the assumption that the detectable difference between the variables should be d = 34.

Therefore, the minimal sample size was estimated at 12.

### Procedure

The exploration test was conducted to assess the exploration of a new environment, the rate of habituation to it, and the response to the introduction of novelty of low intensity into a well-known context. The apparatus and measurement methods were similar to those used in our previous studies^1,12–17^.

**Figure 1:**
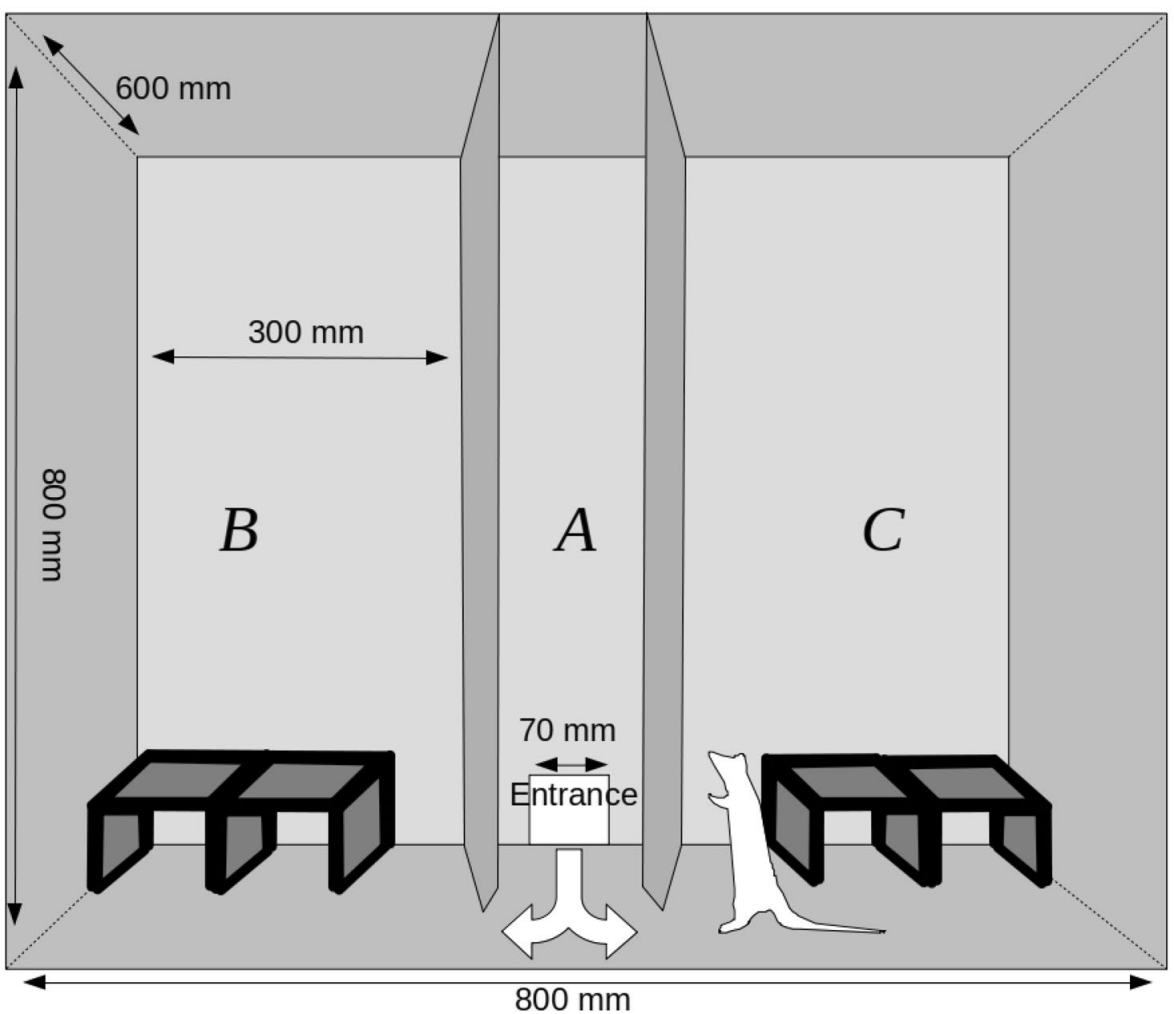
Experimental chamber used for investigating exploratory behaviour.

The experimental chamber (Fig. 1) was a box measuring 800 mm × 600 mm × 800 mm. The chamber was divided into three zones: A, B, and C by two walls running perpendicularly to its longer side. The division walls between the zones had triangular openings (120 mm × 140 mm) at the bottom, which enabled free movement between the chamber parts. There was a hole curved in the back wall of the chamber which served as an entrance for animals going from the transporting device into the chamber. The front of the chamber was made of a transparent plexiglass and it could be lifted to obtain full access to the experimental arena. The entire chamber was covered with a layer of washable varnish. There were tunnels (200 mm × 120 mm × 80 mm) placed in zones B and C made of hard wood covered with washable paint. In contrast to the most frequently used two-dimensional experimental settings, these tunnels provide a complex three-dimensional environment. The central zone (A) was left empty.

**Figure 2:**
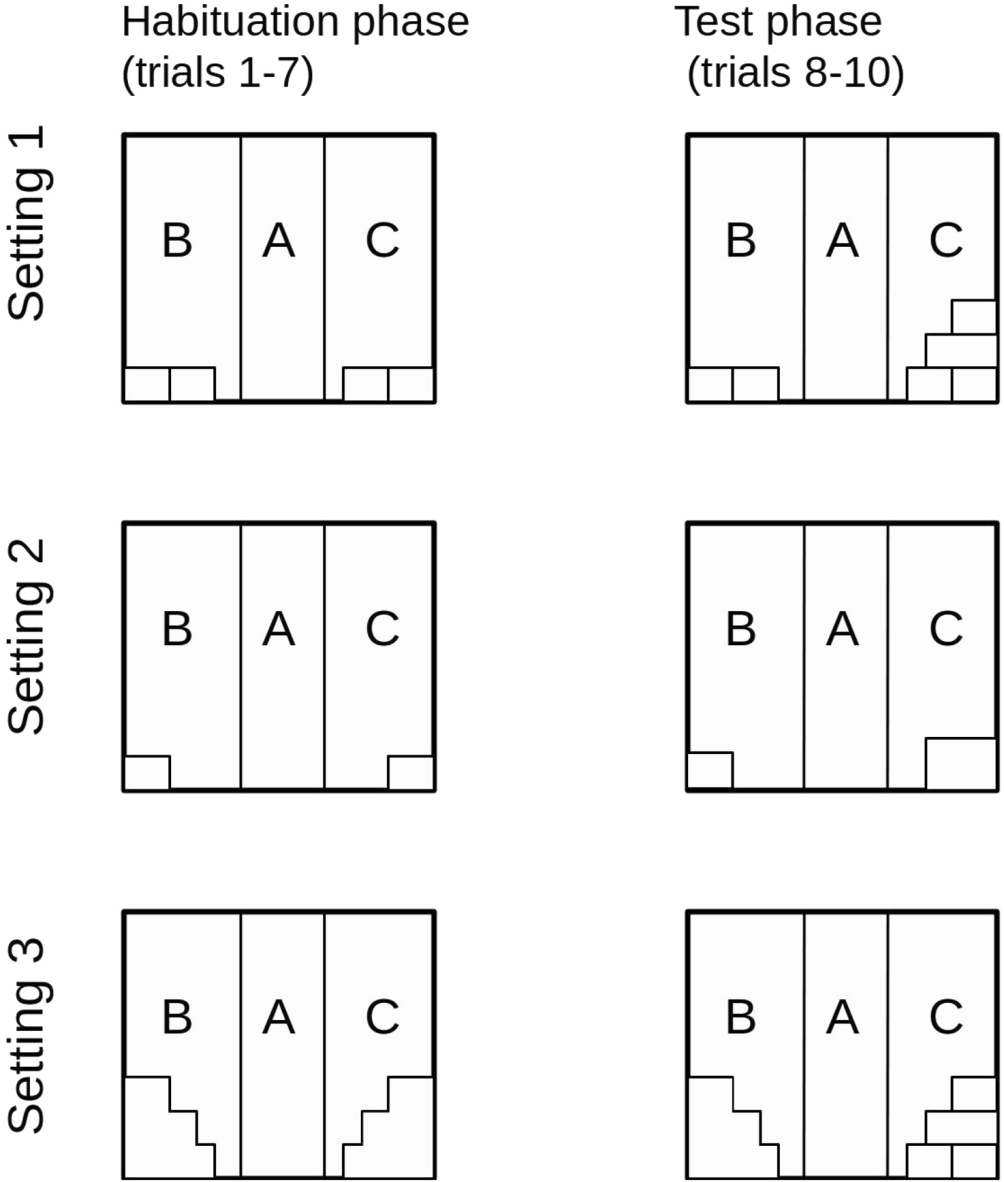
Arrangement of objects in the experimental chamber in each experimental setting.

At the start of each trial, a rat was removed from its home cage and placed in a small cylindrical cage (the ‘transporter’—60 mm in diameter with doors 120 mm high and 100 mm wide). Subsequently, the transporter with the tested animal inside was moved to the experimental room and was placed by the entrance to the zone A. The entrance door was then lifted and it was left open until the end of the trial. The animal was free to stay in the transporter or leave it to explore the chamber. The first seven trials were habituation trials during which the apparatus was arranged in the same way (Fig. 2). The introduction of novelty (i.e. the addition of new tunnels on top of the old ones or the change of the size or type of the tunnels in zone C) took place between trials 7 and 8. The three subsequent trials were conducted with the chamber in this new arrangement (Fig. 2). Each trial was 7 min long and was conducted for each animal once a day. After each session, the experimental arena (including tunnels) and the transporter were thoroughly cleaned with Virkon S (Bayer) in order to remove odour cues left by the previous animal.

A video camera was placed approximately 1.5 m away from the transparent front wall of the experimental chamber. The camera was set in the night-shot mode to enable filming in the dark.

A detailed description of the procedure has been described in a recent paper by Pisula and Modlinska^2^.

The behaviours observed were coded on the basis of the recorded material using BORIS event logging software^18^. The data were sored by well-trained and experienced in animal behavioral analysis PhD students. They had not been informed about the study hypotheses, although they were familiarized with the broad theoretical context. In this study, we scored selected behaviours occurring during the entire experimental session. As a result, the exact time of individual bouts of behaviours, their frequency and, consequently, the total time spent engaging in particular behaviours were assigned specific scores. The behaviours analysed comprised the following: latency to leave the starting box; amount of time spent in the starting box excluding the latency to leave the starting box; total time spent in the unchanged zone of the chamber; total time spent in the changed zone of the chamber; frequency of moving between the zones of the chamber; time spent on contact with the tunnels in the unchanged zone of the chamber; frequency of contact with the tunnels in the unchanged zone of the chamber; total time spent on contact with the tunnels in the changed zone of the chamber; and frequency of contact with the tunnels in the changed zone of the chamber.

Three series of tests were conducted which differed with regard to the configuration of the tunnels that were placed in the experimental chamber, as well as the type of novelty provided in trial 7. In each experimental setting, the tunnels were placed in zones B and C.

#### Setting 1—Addition of a novel object to the other objects in the experimental box—the ADD group

During the habituation sessions, two tunnels 200 mm × 120 mm × 80 mm had been placed in each of the zones B and C and arranged in the same way (Fig. 1). On the first trial day (trial 8), two additional tunnels were put in zone C (Fig. 1). The arrangement of the tunnels in zone B remained unchanged. The ADD group consisted of 14 rats. This setting was a replication of the experimental manipulation applied in the 2019 study^1^. It involved change both in size and complexity of the tunnels in the test arena.

#### Setting 2—Increased size of a familiarized object in the experimental box—the SIZE group

During the habituation sessions, one tunnel was placed in each of the zones B and C (Fig. 1). On the first test day (trial 8), the tunnel in zone C was replaced with an object of the same shape but with an increased size (Fig. 1). No changes were made to the new arrangement in zone C until the end of the experiment. The position of the tunnel in zone B remained unchanged. The SIZE group consisted of 13 rats. This manipulation was applied to create a change in the tunnel's overall size in the test area, but not its complexity.

#### Setting 3—Increased complexity of the objects in the experimental box—the CMPLX group

During the habituation sessions, an object with a height equal to three tunnel diameters, but without internal partitions was placed in each of the zones B and C (Fig. 1). On the first test day (trial 8), the object in zone C was replaced with a construction of the same size but comprising three separate tunnels (with internal partitions)—Fig. 1. No changes were made to the new arrangement in zone C until the end of the experiment. The arrangement of the tunnels in zone B remained unchanged. The CMPLX group consisted of 13 rats. This manipulation was applied to create a change in the tunnel's complexity, but not its overall size.

To avoid the confounding effect of lateralisation or visual/auditory cues, the novelty was introduced in the left zone as described above for half of the rats tested, and in the right zone for the remaining half (a mirror image of Fig. 2).

## Results

To enhance the legibility of the results, the habituation phase was marked as the H mean score from habituation trials 5 to 7, which served as a reference value for further analyses, while the test trials were marked as T1, T2, and T3, respectively. Novelty, i.e. addition or change of objects in zone C, was introduced in the first test trial T1.

The initial four habituation trials have not been presented here, as they served only as a habituation phase and not as an element of the comparative analysis of the animals’ response to novelty.

The data was analysed using a General Linear Model procedure GLM, with repeated measurements H, T1, T2, T3 as within-subject factors, followed by an LSD PostHoc test which involved a comparison of the habituation phase H with the three test trials T1, T2 and T3. Bonferroni correction for multiple comparisons was employed. Differences were considered significant for p ≤ 0.05. Data analysis was carried out using JASP v. 0.14.1 software, an open-source project supported by the University of Amsterdam.

### Time spent in the transporter

The amount of time spent in the transporter, excluding the latency to leave the transporter (that is, the amount of time from the moment the transporter was opened until the rat first entered the experimental apparatus), was measured for each group.

In the ADD group, the analysis showed a significant main effect of trial: F(3, 39) = 5.033, p = 0.005, Eta^2^ = 0.279 (Wilks’ Lambda). A post-hoc analysis showed a significant decrease in the time spent in the transporter in the first and third test trials compared to the habituation phase (T1: p = 0.008, d = 1.090; T3: p = 0.017, d = 0.982).

In the CMPLX group, the analysis showed a significant main effect of trial: F(3, 36) = 8.695, p < 0.001, Eta^2^ = 0.420 (Wilks’ Lambda). A post-hoc analysis showed a significant decrease in the time spent in the transporter in all test trials compared to the habituation phase (T1: p < 0.001, d = 1.564; T2: p = 0.03, d = 1.327; T3: p = 0.027, d = 0.965).

In the SIZE group, the analysis showed a significant main effect of trial: F(3, 36) = 11.934, p < 0.001, Eta^2^ = 0.499 (Wilks’ Lambda). A post-hoc analysis showed a significant decrease in the time spent in the transporter in all test trials compared to the habituation phase (T1: p < 0.001, d = 1.726; T3: p = 0.024, d = 0.986).

### Time spent in the unchanged zone of the chamber

In the ADD group, the analysis showed a significant main effect of trial: F(3, 39) = 15.421, p < 0.001, Eta^2^ = 0.543 (Wilks’ Lambda). A post-hoc analysis showed a significant decrease in the time spent in the unchanged zone of the chamber in all test trials compared to the habituation phase (T1: p < 0.001, d = 3.775; T2: p = 0.043, d = 0.850; T3: p < 0.001, d = 1.441).

In the CMPLX group, the analysis showed a significant main effect of trial: F(3, 36) = 14.825, p < 0.001, Eta^2^ = 0.553 (Wilks’ Lambda). A post-hoc analysis showed a significant decrease in the time spent in the unchanged zone of the chamber in the first test trial compared to the habituation phase (T1: p < 0.001, d = 2.345). Then, a significant increase was observed in the second and third trials compared to the first test trial (T2: p = 0.003, d = 1.291; T3: p = 0.003, d = 1.305).

In the SIZE group, the analysis showed a significant main effect of trial: F(3, 36) = 9.605, p < 0.001, Eta^2^ = 0.445 (Wilks’ Lambda). A post-hoc analysis showed no significant changes in the time spent in the unchanged zone of the chamber between the habituation phase and the first test trial. However, an increase was observed in the second test trial compared to the habituation phase (T2: p = 0.036, d = 0.924). There were also differences between the first test trial and subsequent test trials (T2: p = 0.007, d = 1.176; T3: p = 0.020, d = 1.010).

### Time spent in the changed zone of the chamber

In the ADD group, the analysis showed a significant main effect of trial: F(3, 39) = 21.277, p < 0.001, Eta^2^ = 0.621 (Wilks’ Lambda). A post-hoc analysis showed a significant increase in the time spent in the changed zone of the chamber in all test trials compared to the habituation phase (T1: p < 0.001, d = 2.341; T2: p < 0.001, d = 1.409; T3: p < 0.001, d = 2.092).

In the CMPLX group, the analysis showed a significant main effect of trial: F(3, 36) = 46.825, p < 0.001, Eta^2^ = 0.796 (Wilks’ Lambda). A post-hoc analysis showed a significant increase in the time spent in the changed zone of the chamber in all test trials compared to the habituation phase (T1: p < 0.001, d = 3.634; T2: p < 0.001, d = 2.180; T3: p < 0.001, d = 1.851). Then, a significant decrease was observed in the second and third trials compared to the first test trial (T2: p = 0.020, d = 1.013; T3: p = 0.012, d = 1.088).

In the SIZE group, the analysis showed a significant main effect of trial: F(3, 36) = 32.268, p < 0.001, Eta^2^ =  0.729 (Wilks’ Lambda). A post-hoc analysis showed a significant increase in the time spent in the changed zone of the chamber only in the first test trial compared to the habituation phase (T1: p < 0.001, d = 3.203). Then, a significant decrease was observed in the second and third trials compared to the first test trial (T2: p < 0.001, d = 1.737; T3: p < 0.001, d = 2.294).

### Frequency of moving between the chamber zones (left/right/transporter)

In the ADD group, the analysis showed a significant main effect of trial: F(3, 39) = 5.336, p = 0.004, Eta^2^ = 0.291 (Wilks’ Lambda). A post-hoc analysis showed a significant decrease in the frequency of moving between the zones of the chamber only in the second test trial compared to the habituation phase (T2: p = 0.004, d = 1.201).

In the CMPLX group, the analysis showed a significant main effect of trial: F(3, 36) = 11.567, p < 0.001, Eta^2^ = 0.491 (Wilks’ Lambda). A post-hoc analysis showed a significant decrease in the frequency of moving between the zones of the chamber in the first and second test trials compared to the habituation phase (T1: p = 0.001, d = 1.461; T2: p = 0.003, d = 1.319).

In the SIZE group, the analysis showed a significant main effect of trial: F(3, 36) = 12.171, p < 0.001, Eta^2^  = 0.504. A post-hoc analysis showed a significant increase in the frequency of moving between the zones of the chamber only in the third test trial compared to the habituation phase (T3: p = 0.005, d = 1.214). A significant increase was also observed between the first and third test trials (p < 0.001, d = 1.575) and between the second and third test trials (p = 0.005, d = 1.228).

### Time spent on contact with the tunnels in the unchanged zone of the chamber

In the ADD group, the analysis showed a significant main effect of trial: F(3, 39) = 9.252, p < 0.001, Eta^2^ = 0.416 (Wilks’ Lambda). A post-hoc analysis showed a significant decrease in the time spent on contact with the tunnels in the unchanged zone of the chamber in all test trials compared to the habituation phase (T1: p < 0.001, d = 1.400; T2: p < 0.001, d = 1.413; T3: p = 0.002, d = 1.304).

In the CMPLX group, the analysis showed a significant main effect of trial: F(3, 36) = 11.032, p < 0.001, Eta^2^ = 0.479 (Wilks’ Lambda). A post-hoc analysis showed a significant decrease in the time spent on contact with the tunnels in the unchanged zone of the chamber in the first test trial compared to the habituation phase (T1: p < 0.001, d = 1.836). Then, a significant increase was observed in the second and third trials compared to the first test trial (T2: p = 0.008, d = 1.153; T3: p = 0.007, d = 1.182).

In the SIZE group, Mauchly’s test indicated that the assumption of sphericity had been violated (χ2(5) = 11.860, p = 0.038), so the degrees of freedom were corrected using Greenhouse–Geisser estimates of sphericity (ε = 0.65). The analysis showed a significant main effect of trial: F(1.949, 23.392) = 19.403, p < 0.001, Eta^2^ = 0.618. A post-hoc analysis showed a significant increase in the time spent on contact with the tunnel in the unchanged zone of the chamber in the second and third test trials compared to the habituation phase (T2: p < 0.001, d = 1.674; T3: p = 0.002, d = 1.369). Then, a significant increase was observed in the second and third trials compared to the first test trial (T2: p = 0.002, d = 1.42; T3: p = 0.006, d = 1.185).

### Frequency of contact with the tunnels in the unchanged zone of the chamber

In the ADD group, the analysis showed a significant main effect of trial: F(3, 39) = 5.592, p = 0.003, Eta^2^ = 0.301 (Wilks’ Lambda). A post-hoc analysis showed a significant decrease in the frequency of contact with the tunnels in the unchanged zone of the chamber in the first and third test trials compared to the habituation phase (T1: p = 0.014, d = 1.003; T3: p = 0.005, d = 1.155).

In the CMPLX group, the analysis showed a significant main effect of trial: F(3, 36) = 7.953, p < 0.001, Eta^2^ = 0.399 (Wilks’ Lambda). A post-hoc analysis showed a significant decrease in the frequency of contact with the tunnels in the unchanged zone of the chamber in the first and second test trials compared to the habituation phase (T1: p < 0.001, d = 1.547; T2: p = 0.004, d = 1.249).

In the SIZE group, the analysis showed a significant main effect of trial: F(3, 36) = 3.960, p = 0.015, Eta^2^ = 0.248. A post-hoc analysis showed a significant increase in the frequency of contact with the tunnel in the unchanged zone of the chamber only between the first and third test trials (p = 0.042, d = 0.901).

### Time spent on contact with the tunnels in the changed zone of the chamber

In the ADD group, the analysis showed a significant main effect of trial: F(3, 39) = 25.771, p < 0.001, Eta^2^ = 0.665 (Wilks’ Lambda). A post-hoc analysis showed a significant increase in the time spent on contact with the tunnels in the changed zone of the chamber in all test trials compared to the habituation phase (T1: p < 0.001, d = 3.107; T2: p < 0.001, d = 1.627; and T3: p < 0.001, d = 2.458).

In the CMPLX group, the analysis showed a significant main effect of trial: F(3, 36) = 58.170, p < 0.001, Eta^2^ = 0.829 (Wilks’ Lambda). A post-hoc analysis showed a significant increase in the time spent on contact with the tunnels in the changed zone of the chamber in all test trials compared to the habituation phase (T1: p < 0.001, d = 4.372; T2: p < 0.001, d = 2.101; T3: p < 0.001, d = 2.308). Then, a significant decrease was observed in the second and third trials compared to the first test trial (T2: p = 0.011, d = 1.104; T3: p = 0.008, d = 1.160).

In the SIZE group, the analysis showed a significant main effect of trial: F(3, 36) = 51.999, p < 0.001, Eta^2^  = 0.812 (Wilks’ Lambda). A post-hoc analysis showed a significant increase in the time spent on contact with the tunnel in the changed zone of the chamber in all test trials compared to the habituation phase (T1: p < 0.001, d = 4.757; T2: p = 0.005, d = 1.220; T3: p = 0.012, d = 1.096). Then, a significant decrease was observed in the second and third trials compared to the first test trial (T2: p < 0.001, d = 1.614; T3: p < 0.001, d = 3.428).

### Frequency of contact with the tunnels in the changed zone of the chamber

In the ADD group, the analysis showed no significant main effect of trial: F(3, 39) = 2.169, p = 0.107 (Wilks’ Lambda). In the CMPLX group, the analysis showed no significant main effect of trial: F(3, 36) = 2.098, p = 0.118 (Wilks’ Lambda). In the SIZE group, the analysis showed no significant main effect of trial: F(3, 36) = 2.249, p = 0.099 (Wilks’ Lambda). Table 1 shows the descriptive statistics of all behavioural measures.

**Table 1:** Descriptive statistics of all behavioural measurements analysed in this study.

Group	ADD N = 14		SIZE N = 13		CMPLX N = 13	
Trials	Mean	Std dev	Mean	Std dev	Mean	Std dev
**Time spent in the transporter**						
H	63.119	19.236	69.745	18.948	55.078	15.460
T1	36.143	19.810	44.462	16.831	31.538	17.732
T2	40.571	25.300	42.077	10.555	31.538	14.802
T3	35.214	21.221	49.308	12.828	33.231	17.186
**Time spent in the unchanged zone of the chamber**						
H	131.071	15.306	98.617	21.574	123.025	27.655
T1	67.286	20.140	81.846	27.480	54.846	16.572
T2	97.571	41.598	135.385	36.422	100.385	32.001
T3	83.500	36.044	122.923	29.435	98.462	28.026
**Time spent in the changed zone of the chamber**						
H	119.642	19.156	115.897	28.152	136.282	16.730
T1	221.786	37.579	210.923	19.755	270.538	33.807
T2	213.571	70.028	139.231	35.731	221.692	36.999
T3	228.000	48.651	143.769	24.877	225.077	39.293
**Frequency of moving between the zones (left/right/transporter) of the chamber**						
H	14.334	2.150	16.515	2.999	16.128	3.549
T1	12.500	2.682	14.692	3.449	12.385	2.931
T2	11.429	1.828	14.923	3.303	12.538	2.367
T3	12.714	2.644	19.308	2.983	14.000	3.028
**Time spent on contact with the tunnels in the unchanged zone of the chamber**						
H	81.261	15.945	45.718	11.611	81.205	19.385
T1	46.000	26.452	48.769	17.441	37.846	13.558
T2	50.286	17.495	99.231	30.584	75.077	27.560
T3	47.929	22.54	78.692	23.813	72.000	24.779
**Frequency of contacts with the tunnels in the unchanged zone of the chamber**						
H	6.475	1.211	6.385	1.497	6.538	1.828
T1	4.571	1.828	5.462	2.332	4.385	1.446
T2	4.857	1.994	7.077	2.532	4.615	1.758
T3	4.357	1.277	7.231	1.589	5.154	1.281
**Time spent on contact with the tunnels in the changed zone of the chamber**						
H	71.453	16.395	63.052	21.861	93.768	15.402
T1	170.857	36.538	165.769	18.882	241.692	33.636
T2	164.929	60.431	103.231	30.804	184.846	38.848
T3	184.429	44.284	93.000	23.159	193.769	37.285
**Frequency of contact with the tunnels in the changed zone of the chamber**						
H	5.619	0.949	7.025	1.687	6.488	1.682
T1	7.143	1.748	8.000	1.414	7.077	1.553
T2	6.429	2.709	7.231	2.522	7.923	3.174
T3	6.857	2.214	8.538	2.066	7.923	2.722

#### Effect size analysis

In order to achieve a result analysis design compatible with our previous study (2019), we conducted a similar analysis with regard to the effect size. Table 2 shows all the dependent variables collected in this study across the three experimental groups together with Eta^2^ values. Kruskal–Wallis ANOVA (Eta^2^ value by group) showed no differences between the groups (H = 0.962, df = 2, p = 0.618).

**Table 2:** The ranking list of statistically significant effects based on the partial Eta^2^ values.

Variable	Group	Eta^2^
**Statistically significant effects**		
Time spent on contact with the tunnels in the changed zone of the chamber	CMPLX	0.829
Time spent on contact with the tunnel in the changed zone of the chamber	SIZE	0.812
Time spent in the changed zone of the chamber	CMPLX	0.796
Time spent in the changed zone of the chamber	SIZE	0.729
Time spent on contact with the tunnels in the changed zone of the chamber	ADD	0.665
Time spent in the changed zone of the chamber	ADD	0.621
Time spent on contact with the tunnel in the unchanged zone of the chamber	SIZE	0.618
Time spent in the unchanged zone of the chamber	CMPLX	0.553
Time spent in the unchanged zone of the chamber	ADD	0.543
Frequency of moving between the zones left/right/transporter of the chamber	SIZE	0.504
Time spent in the transporter	SIZE	0.499
Frequency of moving between the zones left/right/transporter of the chamber	CMPLX	0.491
Time spent on contact with the tunnels in the unchanged zone of the chamber	CMPLX	0.479
Time spent in the unchanged zone of the chamber	SIZE	0.445
Time spent in the transporter	CMPLX	0.420
Time spent on contact with the tunnels in the unchanged zone of the chamber	ADD	0.416
Frequency of contact with the tunnels in the unchanged zone of the chamber	CMPLX	0.399
Frequency of contact with the tunnels in the unchanged zone of the chamber	ADD	0.301
Frequency of moving between the zones left/right/transporter of the chamber	ADD	0.291
Time spent in the transporter	ADD	0.279
Frequency of contact with the tunnels in the unchanged zone of the chamber	SIZE	0.248
**Statistically non-significant effects**		
Frequency of contact with the tunnels in the changed zone of the chamber	ADD	0
Frequency of contact with the tunnel in the changed zone of the chamber	SIZE	0
Frequency of contact with the tunnels in the changed zone of the chamber	CMPLX	0

The Eta^2^ values of statistically non-significant effects have been set to “0”.

#### Summary of the results

##### ADD group

The rats from this group responded to the addition of new tunnels with a significant behavioural shift involving increased exploration of the newly installed tunnels. This effect was manifested by a major increase in the time spent in the changed zone of the chamber (in all test trials compared to the habituation phase), as well as the duration of contact with the tunnels in the changed zone of the chamber. What is more, a decrease was observed in the amount of time spent in the unchanged zone, in the duration of contact with the tunnels in this zone and in the length of stay in the transporter (in the first and third test trials; it could be observed in each trial in our previous study). Furthermore, there was a significant decrease in the frequency of moving between the zones of the chamber in the second test trial and in the frequency of contact with the tunnels in the unchanged zone of the chamber. It should be noted that the effects described above were stable across all three test trials. This characteristic is important in the context of comparisons with the other two experimental manipulations.

##### SIZE group

The rats from this group initially reacted with a behavioural shift towards the enlarged tunnel (trial T1) and then towards the unchanged one (trial T2, T3), simultaneously decreasing the amount of time spent in the transporter. This effect was manifested by a significant increase in the time spent in the changed zone of the chamber in the first test trial and in the amount of time spent on contact with the tunnel in the changed zone of the chamber in all test trials (a significant decrease, however, was observed in both cases in the second and third trials). Consequently, the rats from this group exhibited an increase in the amount of time spent in the unchanged zone of the chamber in the second test trial. In addition, there was a significant increase in the time spent on contact with the tunnel in the unchanged zone of the chamber in the second and third test trials. A significant increase was also observed in the frequency of contact with the tunnel in the unchanged zone of the chamber in the third test trial. Moreover, there was a significant increase in the frequency of moving between the zones of the chamber in the third test trial.

##### CMPLX group

The rats from this group exhibited a strong response to the increased complexity of the objects with a profound behavioural shift towards the complex tunnel and then, to a limited extent, towards the unchanged one, with a significant decrease in the amount of time spent in the transporter. This effect was also manifested by a significant increase in the time spent in the changed zone of the chamber, as well as in the time spent on contact with the tunnels in the changed zone of the chamber in all test trials. However, there was a significant (albeit not very steep) decline in the second and third test trials compared to the first test trial (both in the time spent in the zone and on contact with the tunnels). Consequently, a significant decrease was observed in the frequency of contact with the tunnels in the unchanged zone of the chamber in the first and second test trials, in the time spent in the unchanged zone of the chamber in the first test trial (in the case of the latter, an increase was then observed in the second and third trials compared to the first one). Moreover, the rats showed a decrease in the time spent in the unchanged zone of the chamber in the first test trial, and an increase in the second and third trials compared to the first test trial. Furthermore, a decrease was observed in the frequency of moving between the zones of the chamber in the first and second test trials. There was also a significant decrease in the time spent in the transporter in all test trials.

As regards the effect size, no differences were found in any of the three experimental groups, which suggests that all manipulations had similar impact.

## Discussion

It was previously observed^1^ that environmental change involving an increase in environmental complexity triggers exploratory behaviour in a more pronounced way than a change involving the removal or simplification of familiarized objects. However, the manipulation applied in the 2019 study^1^ did not allow us to exclude a concurrent hypothesis that behavioural change observed in that experiment could be explained by the change in the size of the objects modified. The purpose of the present study was to further examine the properties of the stimulus field modification crucial for triggering exploratory behaviour. To achieve this goal, three different types of environmental changes were used as experimental manipulation: the addition of new objects (tunnels), which replicates the manipulation of the 2019 study (ADD); a change in the size of the object (SIZE); and a change in the complexity of the objects (CMPLX). All manipulations applied in the present study involved one-way changes, namely an increase in size and complexity, and not a decrease.

As could be expected based on previous research^1^, all manipulations triggered a positive behavioural shift towards the source of the novel stimulation (modified objects/tunnels) in all test groups. The behaviour of the ADD rats was very similar to the behaviour described in our previous study. The effects of ADD manipulation were very stable across the three test trials. The animals’ activity remained focused on the modified parts of the experimental arena. Therefore, we may confirm that the effects obtained in the 2019 study were replicated in terms of the methodology described by Pisula and Modlinska^2^.

An analysis of the effect size did not reveal any major differences between any of the three manipulations. Since we employed the partial Eta^2^ statistics, which allows us to compare the size of the effects across the experiments and various analyses, the lack of the differences found in this study makes the comparison between this study and the 2019 study interesting. Moreover, a more detailed examination of the results obtained in this follow-up study revealed some interesting manipulation-specific effects.

Changes involving a modification of either object size or object complexity generated different profiles of behavioural response. Although all the rats responded in a very similar way in the first test trial (T1), some differences were observed in the second (T2) and third (T3) trials.

The rats from the SIZE group oriented their behaviour towards the unchanged zone of the chamber in trial T2 and T3, to a similar level as towards the changed one. They also exhibited an increase in the frequency of moving between the zones of the chamber in trial T3. An interpretation of their behaviour should incorporate such concepts as novelty detection^19^, arousal^20^), specific exploration^6,7^, stimulus seeking^21^, and affordances^3,22^. The initial profound response towards the modified part of the test environment clearly demonstrates the animals’ readiness to incorporate novel elements into their cognitive system. The animals undoubtedly did recognise the change and actively reduced the discrepancy between the well-formed representation of a familiar space and the new environmental properties. The animals are in good condition and they are maintained in comfortable conditions. The test arena guarantees a low-stress setting. For this reason, any tasks involving the need to recognise environmental novelty poses no difficulty for them^19^. We may therefore hypothesise that the process of incorporating new information about the changes, with account taken of the simple character of that change (change in size), requires little time and effort. The need for information is satisfied very quickly, but the level of arousal (understood in Hebbian terms)^20^ seems to remain elevated. As demonstrated clearly in a series of studies conducted in 1950s–1970s^6,7,20,21,23,24^, the level of arousal is a key factor regulating exploratory activity and stimulus-seeking. The activational effects of novelty result in an increase in the need for sensory and informational stimulation. However, a simple low-stimulating change in the size of a familiarized object did not provide sufficient stimulation for these needs to be satisfied. Therefore, the animals started to seek stimulation in the other (unchanged) zones of the chamber and generated some additional stimulation by increasing the frequency of moving between all the chamber zones. The validity of this interpretation seems to be borne out by a comparative analysis of the behavioural effects observed in the CMPLX group.

The responses observed in the rats from the CMPLX group must be placed on a continuum between the other two groups. Like in the ADD and SIZE groups, CMPLX rats focused their activity strongly on the changed zone/object in the first test trial (T1). There was a slight change in their behaviour during trials T2 and T3. There was a slight decline of activity in the changed zone in trials T2 and T3. A comparable (slight) increase in activity in the unchanged zone also occurred during trials T2 and T3. These rats also showed a decrease in the time spent moving between the chamber zones in trials T1 and T2, and subsequent increase in this activity in trial T3. It might thus be suggested that a change involving an increase in environmental complexity engaged the animals’ cognitive resources to a greater extent than the change in object size alone. This is an interesting observation from the perspective of the evolutionary theory of cognitive processes. Incorporating high complexity into the cognitive system requires more cognitive resources, and it is measured by analysing the amount of time and effort spent on exploring the more complex object. Exploring a more complex environment creates more opportunities for adaptive behaviours such as escape or foraging. However, it opens a new pathway for the development of individual needs^25,26^, e.g. the capacity to build a more sophisticated cognitive map. This, in turn, is related to novel risks and use of resources.

The 2019 study^1^ demonstrated a clear difference between the behavioural effects triggered by manipulations involving a decrease in the number and complexity of objects vs addition of new objects. The purpose of this study was to address the issue that remained unresolved: the role of complexity vs change in object size in generating a behavioural shift towards the source of novelty (changed objects). The results suggest that two phenomena are at stake. Both an increase in object size and an increase in object complexity trigger a similar process. In fact, the effects of both types of change seem to be cumulative and mutually reinforcing. However, some behavioural effects were observed that were specific to the change in object size or complexity, respectively. The behavioural shift towards the change of the source of stimulus field almost always occurred only in the first test trial (T1). The immediate response allowed the rats to absorb the new information, while some other motivational aspects must have been activated during the next two trials. On the other hand, the change in complexity triggered a longer-lasting response throughout subsequent trials. It is therefore possible that the two environmental manipulations activated different mechanisms of behavioural regulation. These mechanisms may be located on different levels of the complexity/integration hierarchy^27^. The manipulation involving a change in object size may have triggered more fundamental exploration processes consisting in an orienting response and locomotor exploration. An increase in complexity, on the other hand, may have triggered more advanced behavioural activity, such as perceptual exploration and investigatory response^27^. The validation of this hypothesis will require further studies.

From a theoretical standpoint, the data collected supports the view that in rats, curiosity comprises at least two major components: an activational and a cognitive dimension. The activational aspects of curiosity in rats may be explained by novelty-related arousal processes, while the cognitive processes are activated at longer intervals in response to more complex stimulation. In terms of the theoretical framework of ecological psychology^28^, we could say that the more affordances, or the more complex perceptual field, the more advanced the cognitive systems are needed to process them, as Fetterman discussed^29^. An organism’s abilities to exploit affordances offered by the environment and the affordances themselves are not independent from each other. It could therefore be hypothesised that cognitive/motivational mechanisms such as curiosity evolve in close interaction with the environment, which offers certain new opportunities/affordances and excludes others. Further research on these aspects will be conducted with the use of the recently standardized protocol^2^.
